# Analysis of stress distribution around total hip stems custom-designed for the standardized Asian femur configuration

**DOI:** 10.1080/13102818.2014.928450

**Published:** 2014-07-10

**Authors:** Jin Mu Jung, Cheol Sang Kim

**Affiliations:** ^a^ Hemorheology Research Institute, Chonbuk National University, Jeonju, Chonbuk, South Korea; ^b^ Department of Bionanosystem Engineering (BK21 Plus Program), Chonbuk National University, Jeonju, Chonbuk, South Korea; ^c^ Division of Mechanical Design Engineering, Chonbuk National University, Jeonju, Chonbuk, South Korea

**Keywords:** total hip replacement, stress shielding, bone resorption, custom-designed implant stem, stress distribution

## Abstract

In total hip replacement (THR), bone resorption related to the foreign body reaction around the implant causes bonding failure at the bone–prosthesis interface and adversely affects the function and longevity of femoral implants. Stress shielding is thought to be one of the possible biomechanical factors that causes bone resorption, and is related to prosthesis design. We therefore investigated stress distribution at the bone–implant interface of implant models custom-fitted to Asian individuals, using a finite-element method. Based on the standard geometry of Asian femurs, we designed four different custom-fitted implant stems and applied boundary conditions, including a stationary loading of 1750 N. Even though stress shielding was observed for all four different prostheses, the custom-designed implant with a stepped groove in the proximal–medial region had the largest maximum principal stress distribution along paths on the bone–implant interface. This implant type also showed the highest maximum principal stress distribution at the proximal (0.308 MPa), mid (0.872 MPa) and distal (12.981 MPa) regions of the cortical surface of the femur. In conclusion, the implant design with a stepped groove in the proximal–medial region showed an overall increase in stress distribution due to minimization of stress shielding afforded by the reduced effective area in the bone–implant interface. Therefore, this hip implant type could be a possible geometry to remain functional over the long term in THR patients.

## Introduction

Total hip replacement (THR) is a common orthopaedic operation performed to treat patients with degenerative arthritis.[1,2] In THR surgery, the femoral head is cut and removed so that an implant with a long stem can be inserted into the intramedullary canal of the femur. In adhering the implant securely to the femoral canal, a cementless technique has been frequently used for active patients.[3–5] However, despite the long-term stability and functioning of the THR system, several failures of the THR system have been reported.[6]

The principle complication of THR using a cementless fixation is bone resorption around the implant, which results in failure of the bond at the bone–prosthesis interface due to a foreign body reaction between the bone and implant.[7] Stress shielding, a mechanical phenomenon that refers to the reduction of load transferred to the surrounding bone, is one of the possible factors to cause bone resorption.[8,9] After insertion of the implant into the intramedullary canal, the load transferred to the bone could change if the load was partially absorbed by the prosthesis, which in turn would decrease the stress distributed at the bone–implant interface. Shielding of the bone from stress results in bone adaption; there is a metabolic decrease in bone mass with internal or external remodelling, making the bone more porous or thinner as a natural adaptation process corresponding to the decreased carrying load. As a consequence, the bone could become weak and fragile. Such mechanical failure could lead to bone loss or bone resorption and cause stem loosening at the implant-bone interface. Thus, when performing THR, it is important to maintain the pre-operative load transfer (or stress distribution) to the bone to prevent bone resorption.[10,11]

The configuration of the prosthesis has been recognized as an important determinant of stress shielding by defining the contact condition between the implant and bone, and several clinical studies have reported custom-made implant designs to ensure a precise fit in the proximal femur to minimize stress shielding.[12,13] However, even though long-term stability of THR systems has been achieved by varying configuration parameters such as changing the stem size, tapering the stem plane, removing the collar and polishing the prosthesis, most of these approaches were investigated in the general population without considering ethnic differences in the structural geometry of the femur. Structural and morphological differences exist not only among different ages and sexes, but also among different races, i.e. between Caucasians and Asians.[13–15] For example, Khang et al. [16] reported that there were significant differences between Korean and Caucasian femurs in the anteversion angle, the canal flare index, the isthmus cross-section, distance between the lesser trochanter and the isthmus, and shape of the proximal border.

Hence, our aims in this study were to characterize stress distribution around the implanted stems custom designed for THR in Asian individuals. The analysis was performed using a finite-element (FE) method. The optimal design to reduce stress shielding was investigated using four different types of implant stems, suggesting the improved long-term stability of implanted prostheses.

## Methods

### Standard geometry of an Asian femur

To construct a three-dimensional model of a standardized femur, we employed geometric parameters reported in a previous study on the femurs of Korean subjects. In that study, the authors examined computed tomography (CT) images of femurs from 200 healthy Korean subjects (i.e. 100 men and 100 women) without previous trauma. Detailed characteristics of the standard geometry of Korean femurs are described elsewhere.[16]

### Prosthesis designs

**Figure 1.**
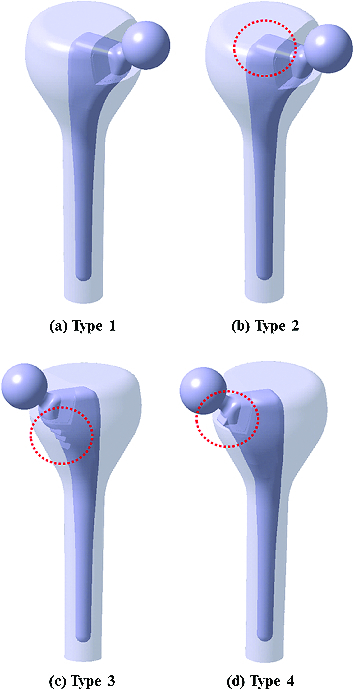
Four different designs of prostheses: type 1 (a) with a standard Korean stem, type 2 (b) with a laterally extended proximal stem, type 3 (c) with a stepped groove in the medial region of the proximal stem and type 4 (d) with a collar added in to the medial section of the proximal stem.

Based on the standard geometry of Korean femurs, a custom-designed implant stem was prepared to investigate stress distribution in the bone–implant interface, as shown in Figure 1(a). Here, the femoral head below the greater trochanter was considered to be cut to insert the implant stem into the femoral canal. The geometric parameters of the implant stems were designed with regard to the anteroposterior and sagittal bone contours of the standard Korean femur. In Figure 1(b)–(d), three different prostheses were additionally designed to analyse the optimal geometry of the implants in terms of stress distribution to the bone. All types of prostheses shown in Figure 1 were assumed to be symmetrically located at the mid-frontal plane of the bone.[10]

The type 1 implant (Figure 1(a)) was the prosthesis without supplementary structures custom-designed for the standard Korean femur. The type 2 implant (Figure 1(b)) was prepared by extending the proximal-lateral region of the custom-designed prosthesis. Types 3 and 4 (Figure 1(c) and 1(d)) were designed by adding a stepped groove and a collar, respectively, to the proximal–medial area of the custom-designed prosthesis. Note that types 2, 3 and 4 were supplemented with additional structures within the allowable range of the proximal area for THR surgery. The prostheses were considered to be made of corrosion-resistant stainless steel. The applied Young's moduli for cancellous and cortical bones were 728 MPa and 17 GPa with the same Poisson's ratios of 0.3, respectively. The elastic modulus, the tensile strength and Poisson's ratio of the implant were assumed to be 200 GPa, 480 MPa and 0.3, respectively. The bending strength of the implant was applied as twice the tensile strength (i.e. 960 MPa). The possible chemical composition [17] of the implanted stem was considered to be Cr (18.00 wt %), Ni (12.00 wt%), Mo (2.50 wt%), Mn (1.70 wt%), Si (0.15 wt%), P (0.04 wt%), S (0.01 wt%), C (0.02 wt%) and Fe (balance), respectively. The materials used in the present study were assumed to show linear elastic and isotropic behaviours.

### Finite-element model, loading and boundary conditions

Three-dimensional models of the standardized Korean femur and four types of prostheses were constructed and the structural characteristics of the bone and implants in response to loading were analysed using Ansys Workbench FE software. FE models were constructed with 471,445, 473,770, 475,488 and 468,126 elements for type 1, 2, 3 and 4 prostheses, respectively.

**Figure 2.**
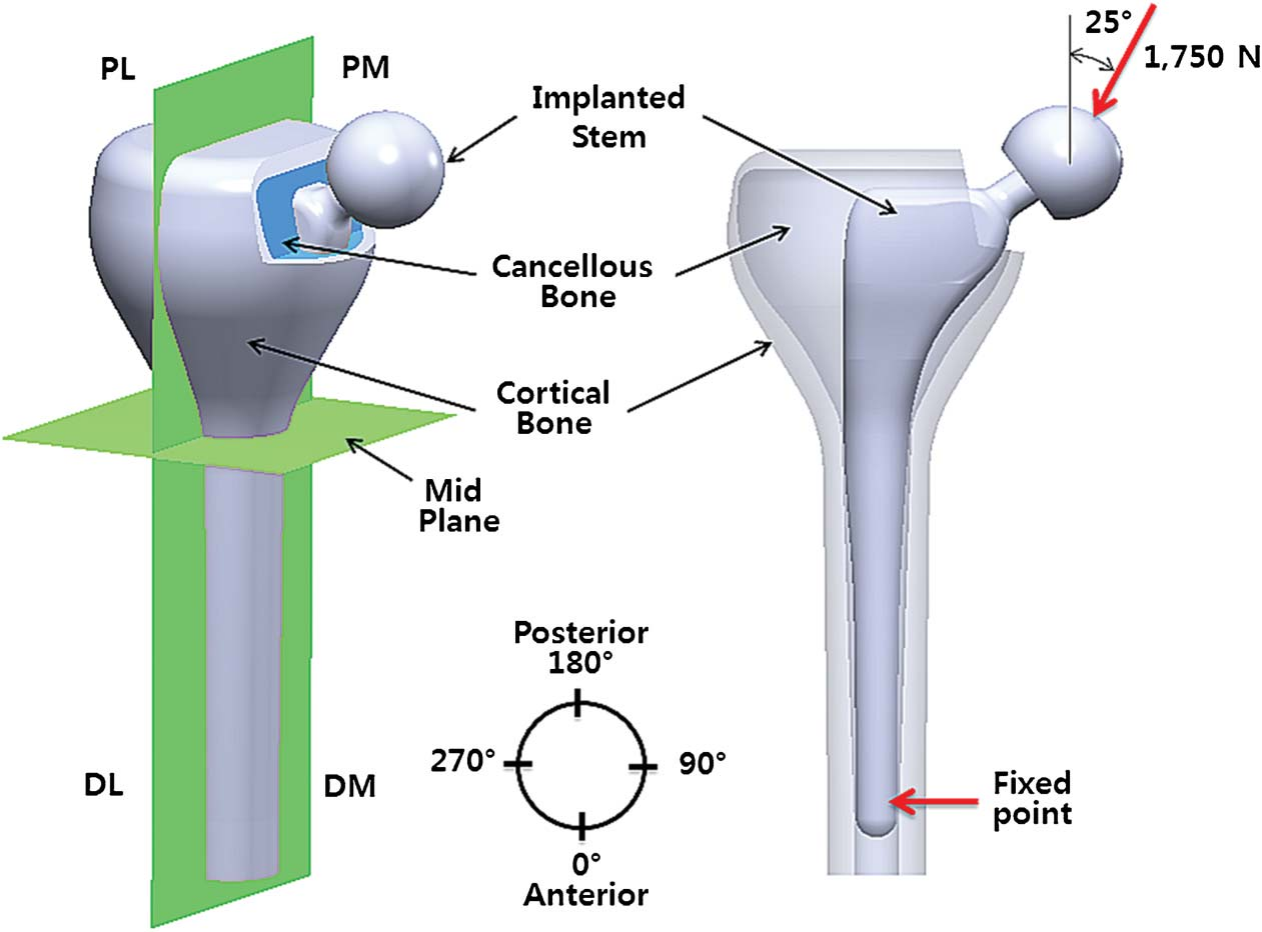
Schematic descriptions of the implanted stem, cancellous bone and cortical bone under a constant loading condition. PL, PM, DL and PM indicate proximal-lateral, proximal–medial, distal–lateral and proximal–medial areas of the femur, respectively.

Based on the following assumptions, boundary and loading conditions were applied to the FE models. The implant stems inserted into the intramedullary canal were adhered to the bone using a cementless technique to securely hold the implant in the femoral canal, as shown in Figure 2. No slipping occurred among elements such as the femoral stem, cancellous bone or cortical bone. The FE models of the implant stems were fixed to the bone at 15 mm from the bottom of the stem. A simplified stationary loading of 1750 N was applied to the head at an angle of 25° from the straight line to the femoral axis direction, matching the loading configuration of a person with a body weight of 70 kg standing on one leg. Note that we reasoned that standing is the second most frequent activity during the day of a patient (sitting is the most frequent activity).[18]

As shown in Figure 2, the femur was divided into four separate regions, proximal–lateral, proximal–medial, distal–lateral and distal–medial areas, for consistent description. Considering the brittleness of bones and the elasticity of implant, stress distribution was expressed with regard to the maximum principal stress and von Mises stress. The prostheses inserted into the intramedullary canal were of particular interest in the present study.

## Results and discussion

### Maximum principal stress at the bone–implant interface

**Figure 3.**
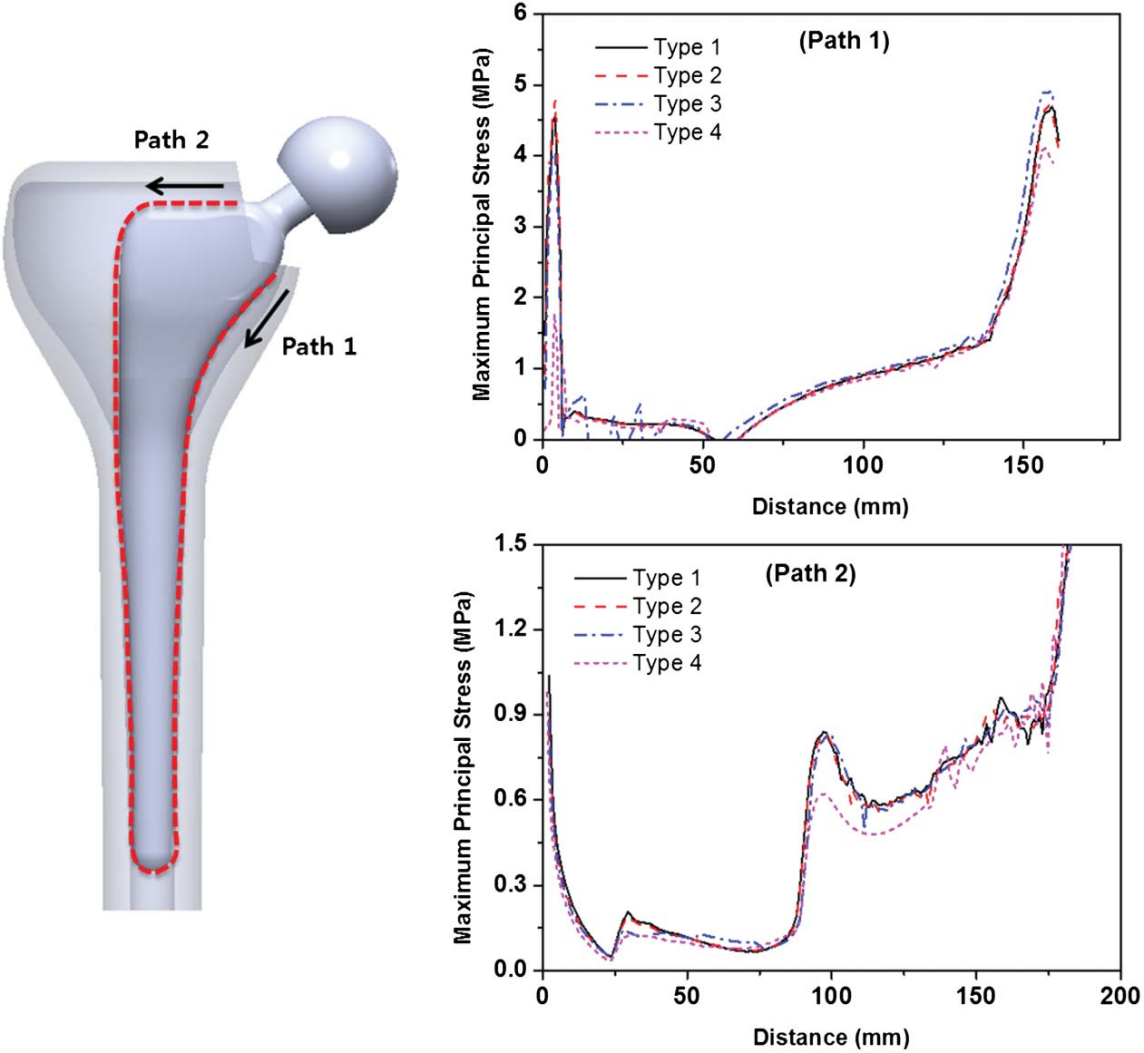
Maximum principal stress distribution along the bone–implant interface under a constant loading of 1750 N.

Figure 3 shows the distribution of maximum principal stress along the length of the bone–implant interface of the femur when a constant loading of 1750 N was applied to the four different types of prostheses. In general, maximum principal stress increased along the paths 1 and 2 from the proximal region to the distal region of the femur, with the peak value observed at the distal region of the implant stem. For path 1, stress distribution of type 3 increased by 1.6% on average when compared to type 1 along the full length. The type 4 implant had the lowest stress distribution among the four types of prostheses, showing an average 19.0% reduction when compared to type 1 along the full length. For path 2, an overall reduction in stress distribution from proximal to distal regions was also observed for type 4. Stress distribution was reduced by 8.0% on average when compared to type 1. However, the other types (i.e. types 2 and 3) experienced no significant differences in stress distribution.

The peak value of maximum principal stress (up to about 40 MPa) was observed at the tip of the prosthesis stem; this artificial phenomenon could be due to our use of a fixed boundary condition. The gradational increase in maximum principal stress along the length of the implant was related to bending caused by oblique application of a load to the implant head. For the type 4 implant, the reduced stress distribution observed along paths 1 and 2 could be caused by the absorbed loading at a collar. This low stress distribution could potentially enhance stress shielding and lead to further bone resorption by weakening the bone. In contrast, for the type 3 implant, the increased stress distribution along path 1 could be due to the reduced interacting area of a stepped groove. Even though the stepped groove geometry had a larger surface area than type 1, the effective load-transferring area could be reduced due to a line contact contour of the stepped groove to bone. The reduced effective area in the bone–implant interface might have increased stress distribution to the bone.

### Maximum principal stress around the surface of cortical bone

**Figure 4.**
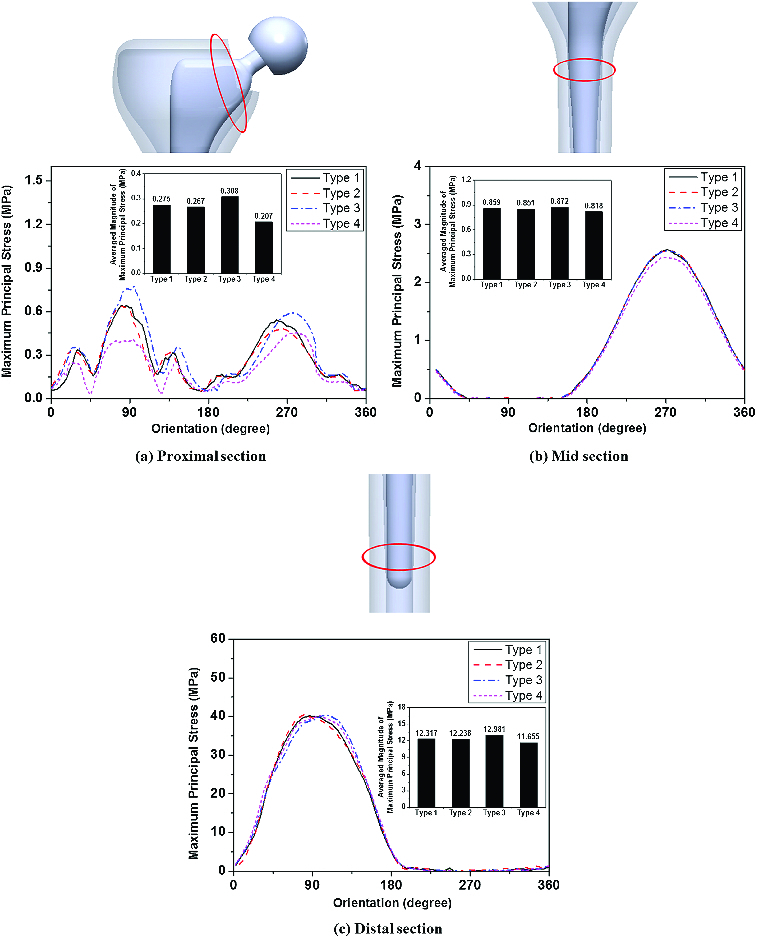
Maximum principal stress distribution around the cortical surface of proximal (a), mid (b) and distal (c) areas of the femur.

Figure 4 shows the maximum principal stress around the cortical surface at the proximal, mid and distal sections of the femur for the four different types of prostheses. Overall, the stress magnitude increased at orientation angles of 90° and 270° due to bending. In Figure 4(a), the type 3 implant showed the largest increase in the stress magnitude (0.77 MPa) at the proximal–medial region, while the lowest stress value (0.40 MPa) was observed in the type 4 implant at this region. As shown in Figure 4(b) and 4(c), all four types of prostheses showed similar variations in the change of stress magnitude at the mid and distal regions. Interestingly, when comparing the average stress values around the cortical surface, there was an overall increase in stress for the type 3 implant. It was found that type 3 had the largest values at the proximal (0.308 MPa), mid (0.872 MPa) and distal (12.981 MPa) regions, respectively. In contrast, the type 4 implant showed the lowest average stress values of 0.207 MPa, 0.818 MPa and 11.655 MPa at these regions, respectively.

The load transferred to the bone needs to be maintained at pre-surgical levels to prevent bone resorption of the femur. This can be achieved by modifying the biomechanical parameters of the implant, such as stress distribution. Bone resorption around hip stems can cause the bone to become thinner or more porous, which is a natural adaptation process of the bone to the reduced load. This loss of bone mass could reduce the fixation strength of the remaining bone to the implant to a level where the implant might not be strong enough to withstand external loading.[10]

### Equivalent stress along the cross-sections of the femur

**Figure 5.**
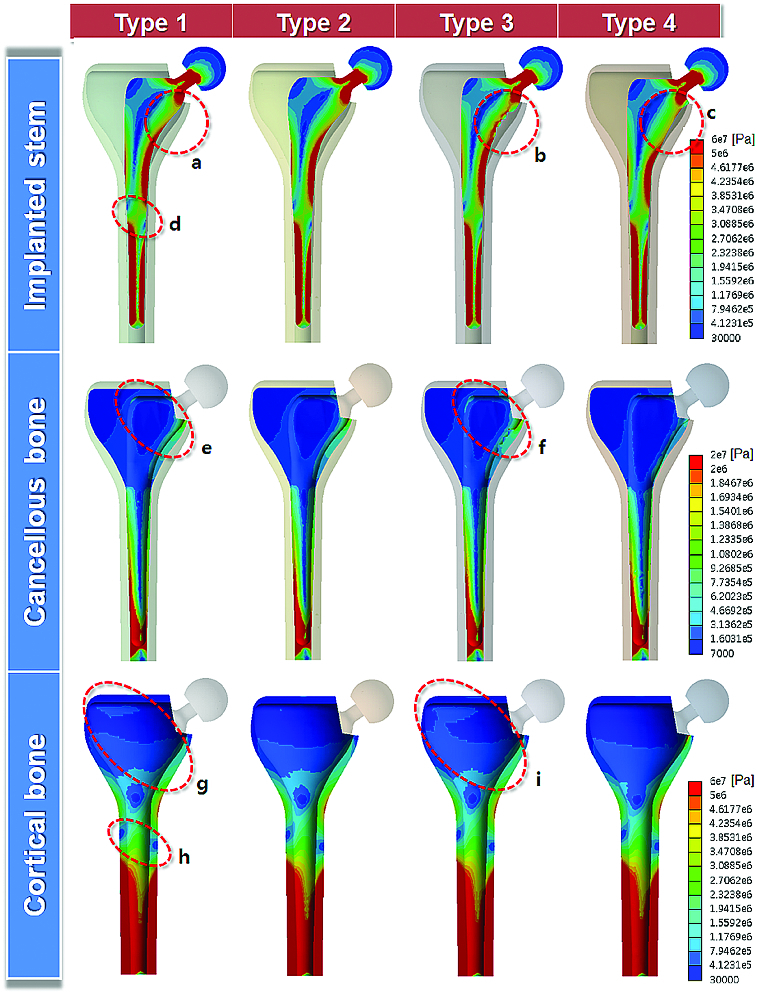
Equivalent stress distribution at the cross-section along the length of the implanted stem, cancellous bone and cortical bone.

Figure 5 shows the distribution of von Mises stress (equivalent stress) along the longitudinal cross-sections of the implanted stems, cancellous bones and cortical bones. Stress distributed along the length of the femur generally increased towards the distal region, as mentioned previously (see Figures 3 and  4). No significant difference was observed among the four types at both the distal–lateral and the distal–medial regions. However, the prostheses with supplementary structures showed particular difference in stress distribution. The response of the implanted stems to external loading was described in the first row in Figure 5. Stress distributed in the type 3 implant increased at the proximal–medial region (circle b) when compared to the custom-designed prosthesis (the type 1 implant; circle a). However, stress in the type 4 implant was reduced at this region (circle c). Different load-transferring conditions for the four implanted stems resulted in different stress distribution in cancellous bones, as shown in the second row in Figure 5. When compared to type 1 (circle e), type 3 showed a greater magnitude in stress distribution at both the proximal-lateral and the proximal–medial regions (circle f). In the third row in Figure 5, stress distribution along the cross-sections of cortical bone showed a similar tendency to that observed for the cancellous bone, but the stress was more evenly distributed than the cancellous bone in both type 1 (circle g) and type 3 (circle i) because the load was propagated through the thickness of the bone.

Overall, the type 3 implant showed the largest stress distribution at the longitudinal cross-sections of the femur, indicating the lowest stress shielding effect of all four implant types. The stress distribution at the cross-section of the type 2 implant was also analysed, but the difference from type 1 was not significant, possibly because the extended geometry in the proximal–lateral region of type 2 did not influence the load transfer. Interestingly, in the mid-plane area of the femur, a sudden decrease in stress distribution was commonly observed at both lateral and distal sides (circles d and h), where frequent failure of the implant in this region has been clinically reported.[19,20]

Stress distribution in the bone–implant interface is determined primarily by the effective area of the implant. A change in the effective area of the interface would affect stress distribution to the bone, because stress is a function of load and area.[11] For these reasons, the type 3 implant, which had a stem with a stepped groove in the proximal–medial region, might have transferred higher stress to the bone in the most desirable way because of the reduced effective area in the bone–implant interface, resulting in a possible reduction in stress shielding and thus enhancement of bone growth. These hypotheses should be investigated in future clinical studies, in which bone remodelling patterns need to be analysed by monitoring the density change of bone after the initial stimulus and during remodelling using imaging techniques.

## Conclusions

The extent of stress shielding caused by the implanted stem was investigated using four different designs of prostheses custom-designed for the standard Korean femur. The maximum principal stress at the bone–implant interface increased gradually along the length of the implant due to the bending effect. The type 3 implant, which had a reduced interface area due to the presence of a stepped groove at the proximal–medial region of the implant, showed a higher stress distribution in the bone–implant interface than the other implant types. Around the cortical surface, the type 3 implant showed the largest increase in the average stress magnitude at the proximal, mid, and distal regions. Along the longitudinal cross-sections of the implant, cancellous bone, and cortical bone, the type 3 implant presented an overall increase in stress distribution throughout the entire length compared to the other stem configurations. However, the type 2 implant had the lowest stress distribution, most likely due to the reduced load transfer taken by a collar. The type 3 implant with a stepped groove in the proximal–medial region of the stem yielded the most desirable result of low stress shielding, which may have been due to a reduction in the effective area at the bone–implant interface. 
